# VIS-XUV Optical Constants and Electronic Band Parameters of a Tin-Oxo Cage Photoresist (Sn_12_O_24_C_52_H_120_)

**DOI:** 10.1021/acs.jpcc.5c06405

**Published:** 2025-10-31

**Authors:** Wolfgang S. M. Werner, Florian Simperl, Felix Blödorn, Olga Ridzel, Quentin Evrard, Albert M. Brouwer

**Affiliations:** † Institut für Angewandte Physik, 27259 Technische Universität Wien, Wiedner Hauptstraße 8−10,/E134 A-1040 Vienna, Austria; ‡ Theiss Research, 7411 Eads Ave., La Jolla, California 92037-5037, United States; § Van ’t Hoff Institute for Molecular Sciences, 1234 University of Amsterdam, P.O. Box 94157, 1090 GD Amsterdam, The Netherlands

## Abstract

Energy band parameters and VIS-XUV optical constants were measured on a Tin-Oxo cage photoresist by employing reflection electron energy loss spectra (REELS) and secondary electron–electron energy loss spectroscopy (SE2ELCS). Different kinematic conditions were chosen for the two reflection loss spectra in order to disentangle contributions of volume and surface losses. The normalized differential inverse inelastic mean free path (nDIIMFP) was extracted and fitted to a model dielectric function, described as a sum of Drude-type oscillators. The oscillator parameters were used to calculate the energy loss function (ELF) and the electron inelastic mean free path (IMFP). An energy gap of *E*_g_ = 6.6 ± 0.5 eV was determined from the onset of the energy loss function. The energetic distance between the valence band maximum (*E*_VBM_, or highest occupied molecular orbital, HOMO) and the vacuum level (*E*_vac_) was established by electron-pair spectroscopy by measuring the smallest energy loss leading to emission of a secondary electron. This was found to be 9.9 ± 0.5 eV, giving the electron affinity as χ = *E*_VBM_ – *E*_vac_ – *E*_g_ = 3.3 ± 0.8 eV. The valence bandwidth was estimated from the coincidence data to be *E*_v_ = 8.5 eV.

## Graphical abstract

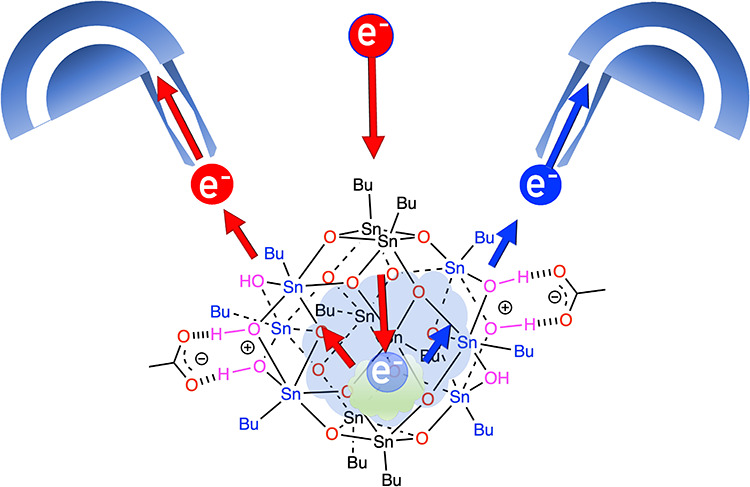

## Introduction

Extreme ultraviolet (EUV) photolithography, using EUV photons with a wavelength of 13.5 nm (92 eV), nowadays makes it possible to manufacture features in microelectronics with dimensions below 10 nm.^1,2^ The use of photons with elevated energies also calls for changes in the manufacturing process, in particular the photoresist material, since (1) the photoionization cross section of organic photoresists at EUV energies is strongly reduced compared to, e.g., 193 nm immersion photolithography (*hν* ∼ 6 eV), and (2) most importantly, the mechanism of energy dissipation of EUV-induced photoelectrons implies an increased diffusion of the active agents, low-energy electrons produced by inelastic photoelectron scattering, responsible for the desired photoconversion. This leads to a blurring of the developed patterns.

Presently, photoresist research for EUV is exploring a wide variety of molecular materials, including organic, inorganic, and hybrid compounds.^3–8^ Among the earliest prototypes for metal–organic resists are the tin-oxo cages, which are related to commercial EUV resists.^9–11^ In the present work, we investigate thin films of a butyltin-oxo cage with acetate counterions (Figure 1).

**Figure 1:**
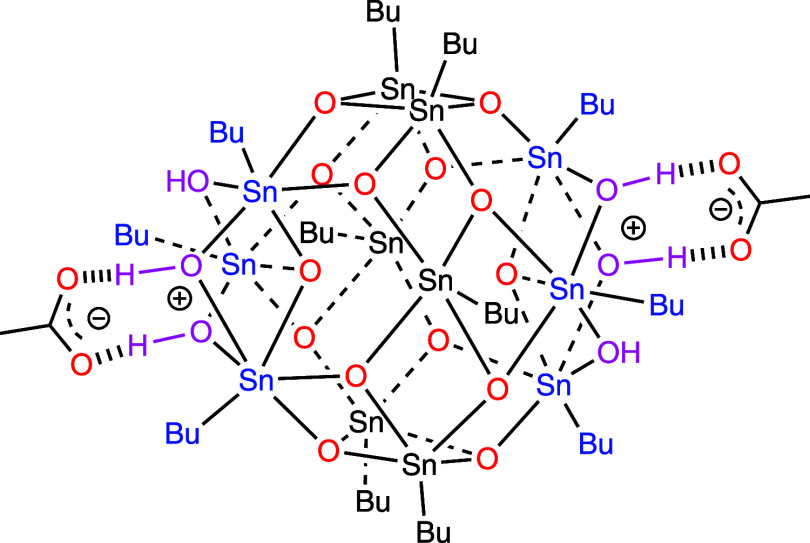
Chemical structure of investigated *n*-butyltin-oxo cage photoresist with acetate (CH_3_COO^–^) counterions.

The ionization cross section of Tin peaks near 92 eV,^12^ making tin-oxo cages a good choice for a resist material. In the course of plural or multiple inelastic scattering processes, photoelectrons repeatedly liberate low-energy electrons from the solid, which eventually dissipate the incoming energy. The trapped electrons and the holes that are simultaneously formed are chemically reactive sites for the photoconversion process.^13^ The extent of the volume in which photoelectrons and liberated solid-state electrons occur is determined by the susceptibility of the solid-state electrons to become polarized by an external perturbation, since this determines the average path length traveled between subsequent excitations.

In the above context, the availability of optical constants over a wide spectral range, from the VIS regime to the EUV regime, is of paramount importance. In the EUV regime, this concerns the absorption of the incoming light, which can be quantified for any material using the data compiled by Henke.^14^ For understanding the energy dissipation of the primary photoelectrons and the low-energy electrons liberated by inelastic photoelectron scattering, optical constants in the VIS-XUV regime are mandatory to evaluate the electron inelastic mean free path (IMFP) and related quantities using linear response theory. For arbitrary materials, such data are not readily available.

In the present work, VIS-XUV optical data of a Tin-Oxo cage photoresist are determined by measuring reflection electron energy loss spectra (REELS), which are subsequently analyzed by means of a well-established procedure giving the dielectric function.^15–17^ Furthermore, the band parameters, in particular the energy gap, *E*_g_, and electron affinity, χ, as well as the width of the valence band, *E*_v_, were determined using a combination of single electron and electron-pair spectroscopy.^18,19^

## Experimental Section

Silicon substrates, 1 × 1 cm^2^ in size, were cleaned with a base piranha solution heated to 80 °C for 15 min, rinsed with isopropanol, and dried with nitrogen flow. Before use, the substrates are further cleaned using a low-pressure oxygen plasma cleaner (Diener Electronic Pico GR-200-PCCE) with a 2 min oxygen plasma with a 0.2 mbar working pressure. A 12 mg mL^–1^ solution of the tin-oxo cage with acetate counterions (synthesized according to Eychenne-Baron et al.^20^) is prepared in methyl isobutyl ketone and sonicated for 10 min. The solution is filtered through a 0.2 μm PTFE filter and spin-coated directly on the cleaned silicon substrates at 2000 rpm with a 750 rpm/s acceleration and 45 s spin-coating time. The samples are placed inside a glovebox and sealed in a nitrogen atmosphere. The resulting films have an RMS roughness of 0.4 nm and a thickness of approximately 25 nm as assessed by means of atomic force microscopy. Table 1 summarizes the material parameters of the investigated tin-oxo cages.

The Tin-Oxo cage samples were received from UvA in airtight sealed sample storage boxes and introduced within a minute in the secondary electron–electron energy loss coincidence (SE2ELCS) apparatus at the TU Wien, consisting of a Kimball physics ELG-2 Electron gun, an electrostatic hemispherical analyzer (HMA), and a home-built time-of-flight (TOF) analyzer, a drift tube, and a detector comprising a multichannel plate stack and a delay line anode. These components are mounted in a UHV system where the pressure during the measurements is typically 2 × 10^–10^ mbar (for details, see the SM of ref 21).

Single electron spectroscopy is performed in the usual manner by scanning the energy in the hemispherical analyzer; for electron-pair spectroscopy, the arrival times of electrons in both analyzers, the TOF and HMA, are written to disk, and a histogram of flight time differences retrospectively yields the coincidence spectrum.^21^ While the TOF analyzer detects electrons with any energy in parallel, the HMA only accepts those with a preset energy. Scanning the energy of the HMA and measuring a coincidence spectrum at every preset then yield the double differential coincidence spectrum discussed below. The net time resolution on the order of a nanosecond is governed by the flight time spread of trajectories on their Kepler orbits in the HMA.

Reflection electron energy loss spectra (REELS) were measured using the electrostatic analyzer at a pass energy of 20 eV (0.5 eV energy resolution). In order to be able to disentangle contributions of surface and volume scattering two spectra were taken at conditions corresponding to high surface sensitivity (primary energy *E*_0_ = 300 eV, incidence angle θ_*i*_ = 80°, and emission angle θ_0_ = 40°, both w.r.t. the surface normal) and high volume sensitivity (*E*_0_ = 1600 eV, θ_*i*_ = 60°, θ_0_ = 60°).

An Auger-Meitner spectrum was measured (at 1.6 keV primary energy) and quantified using the peak-to-peak heights in the derivative spectrum and tabulated atomic sensitivity factors (for 3 keV). This resulted in ratios of atomic concentrations of Sn/C = 0.21 and Sn/O = 0.71, reasonably close to the expected values of Sn/C = 0.23 and Sn/O = 0.5, indicating a tendency of the surface to become metallic under electron bombardment but at the same time ruling out a serious surface contamination.

Coincidence measurements were performed with a primary energy of 1000 eV and a pass energy of 200 eV. The incident current during the coincidence measurements is about 0.4 fA, and the sample position was changed by 1 mm every 24 h in order to minimize charging: for the focus of the electron gun of several hundred μm, such conditions imply that each atom on the surface, on average, sees at the most one incoming electron. The coincidence measurements are therefore believed to constitute a very sensitive measurement of the surface.

**Table 1:** Material Parameters of the Investigated Photoresist (Sn_12_O_24_C_52_H_120_)­^a^

	*Z*_av_	ρ (g/cm^3^)	*n*_a_ (nm^–3^)	*E*_g_ (eV)	χ (eV)	*E*_v_ (eV)	*n*(0)
TinOAc	5.88	1.9	93	6.6	3.3	8.5	1.54

a Average atomic number *Z*_av_, mass density *ρ*,^22^ atomic density *n*_a_, band gap *E*_g_, electron affinity *χ*, width of the valence band *E*_v_, and static refractive index *n*(0) derived from the present results.

## Results and Discussion

The blue curves in Figure 2 show the energy loss spectra after performing a Lucy-Richardson deconvolution^23^ to eliminate the broadening due to the energy spread of the electron gun and from which the elastic peak was subtracted after fitting it to a Gaussian. Division of the resulting spectrum by the area of the elastic peak gives the loss spectra in absolute units of reciprocal eV,^15^ as shown in Figure 2. The REELS spectra are a superposition of multiple self- and cross-convolutions of the distribution of energy losses in a single surface and volume scattering process, given by differential surface excitation probability (DSEP) and differential inverse inelastic mean free path (DIIMFP). Surface excitations are a consequence of the boundary conditions of Maxwell’s equations at interfaces with different dielectric constants and appear in a depth range of about a monolayer about the surface, both in vacuum and inside the solid. Volume excitations take place over a depth range of a multiple of the electron inelastic mean free path. The single scattering loss distributions were retrieved by simultaneous deconvolution of the two REELS spectra according to the procedure in refs 15,16 . The resulting (normalized) DIIMFP is presented in absolute units of reciprocal eV in Figure 3a as the blue curve.

**Figure 2:**
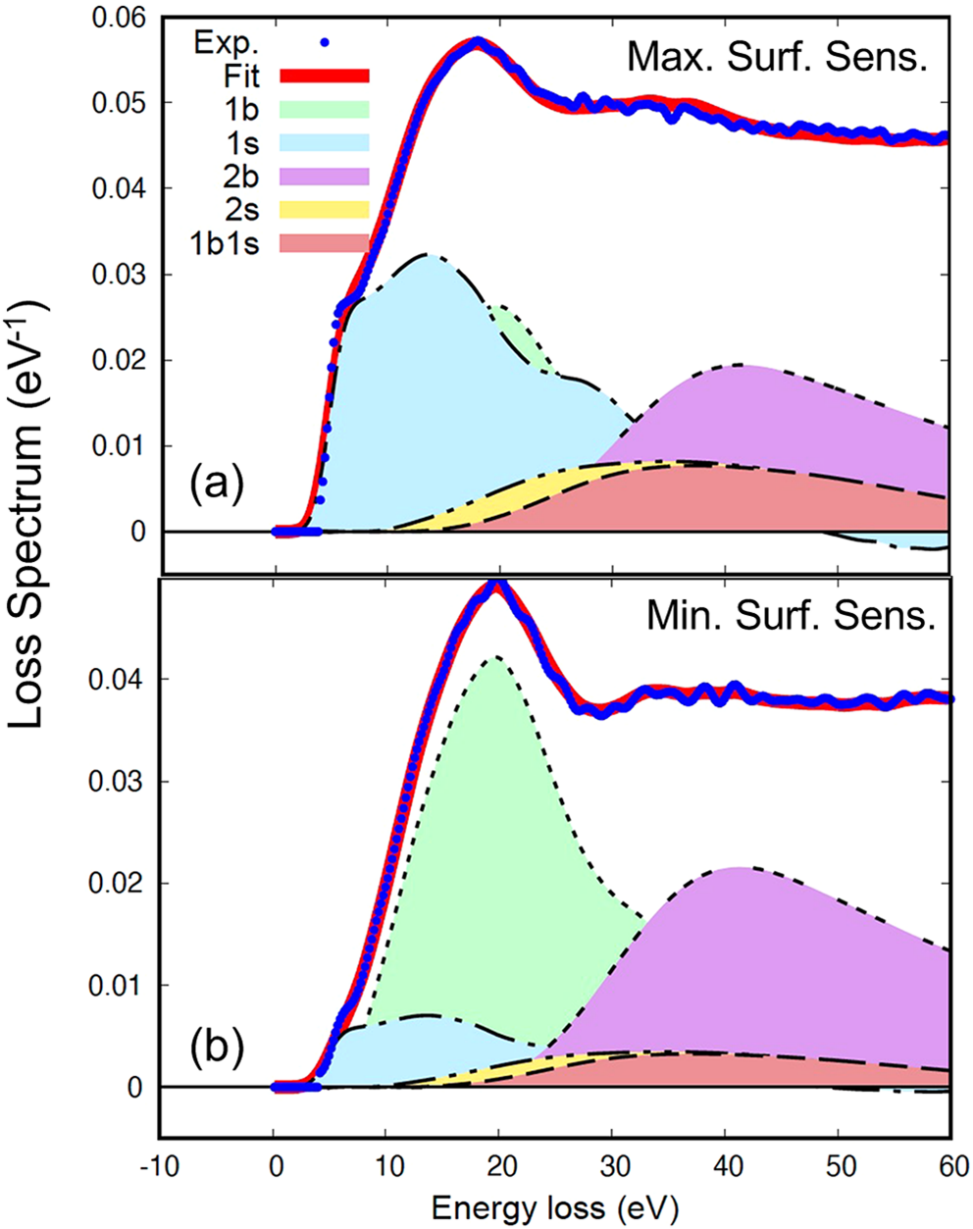
Blue curve: raw experimental data after Lucy-Richardson deconvolution of the broadening due to the finite width of the elastic peak. Red curve: fit of the raw REELS-data to a superposition of the multiple self- and cross-convolutions of the DIIMFP and DSEP; filled colored curves: multiple scattering contributions; b—bulk (DIIMFP), s—surface (DSEP). (a) Kinematical conditions corresponding to greater surface sensitivity and (b) smaller surface sensitivity.

**Figure 3:**
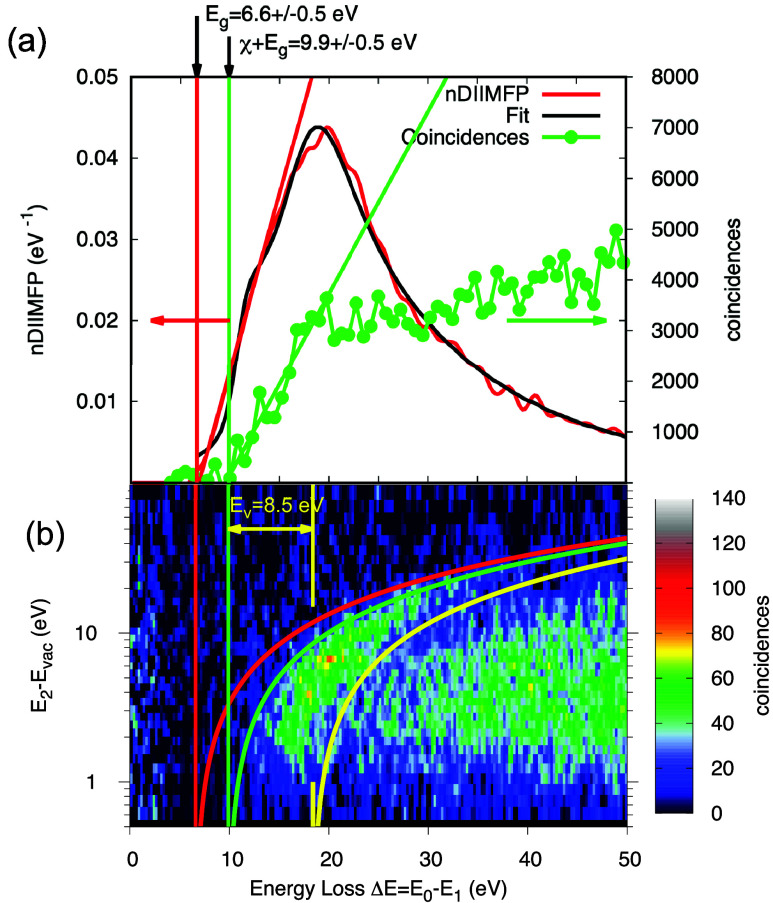
(a) Red curve: normalized DIIMFP retrieved from the loss spectra; black curve: fit of the nDIIMFP to eq 1; green curve: sum of the double differential coincidence data in (b); the green and red vertical lines indicate the onset of bulk losses and coincidences, respectively. (b) Double differential secondary electron–electron energy loss coincidence spectrum (SE2ELCS).

Figure 2 also shows the decomposition of the REELS into a linear superposition of contributions of electrons that have participated in a certain number of bulk (label “b” in Figure 2) and surface (label “s”) excitations. The red curve represents the sum of all contributions, and the filled colored curves labeled “1b”, “1s”, “1b1s”, etc., indicate the individual contributions of particles arriving in the detector after a given number of bulk and surface excitations in the solid. These contributions are given by multiple self- and cross-convolutions of the DIIMFP and DSEP, weighted with the relative number of electrons detected after a certain number of bulk and surface losses.^15,24^ The spectrum taken at maximum surface-sensitive conditions is seen to be dominated by surface excitations for energies <20 eV (panel (a), blue filled curve), which are strongly reduced in the “min” spectrum in panel (b). The different surface sensitivities of the two REELS spectra can also be inferred from their shape in that the shoulder at Δ*E* ≈ 4 eV is more pronounced in the “max”-spectrum and the “min” spectrum has a local minimum at around 30 eV, which cannot be discerned in the “max”-spectrum.

Finally, the optical constants were determined by fitting the nDIIMFP retrieved by deconvolution of the two REELS to a model dielectric function.^16^ This is done by considering the relationship between the dielectric function and the probability for an electron with primary energy *E* to lose energy ω in an individual inelastic scattering event accompanied by a momentum transfer *q* inside the bulk of a solid, the so-called bulk differential inverse inelastic mean free path (DIIMFP) *W*_in_ (ω, *E*). This quantity is given by^25,26^

1 Win(ω,E)=(1+E−Egc2)21+E−Eg2c21π(E−Eg)∫q−q+Im[−1ε(ω,q)]dqq

with

2 $$q_{\pm }=\sqrt{(E-E_{g})\left(2-\left(E-E_{g}\right)/c^{2}\right)}\pm \sqrt{(E-E_{g}-\omega )\left(2-\left(E-E_{g}-\omega \right)/c^{2}\right)}$$

where Im [−1/ε (ω, *q*)] is the energy loss function (ELF), *E*_g_ is the bandgap energy, and *c* denotes the speed of light. Here and below, atomic units are used. The dielectric function ε (ω, *q*) is approximated by an extended Drude model dielectric function in terms of a set of oscillators with amplitudes or oscillator strengths *A*_*i*_, binding energies ω_*i*_, and damping coefficients Γ_*i*_ ^27^

$$\varepsilon_{1}=\varepsilon_{b}-\sum_{i}\frac{A_{i}\;\left(\omega^{2}-\omega_{i}\;{\left(q\right)}^{2}\right)}{{\left(\omega^{2}-\omega_{i}\;{\left(q\right)}^{2}\right)}^{2}+\Gamma_{i}^{2}\omega^{2}}$$

3 $$\varepsilon_{2}=\sum_{i}\frac{A_{i}\Gamma_{i}\omega }{{\left(\omega^{2}-\omega_{i}\;{\left(q\right)}^{2}\right)}^{2}+\Gamma_{i}^{2}\omega^{2}}$$

with ε_b_ being the background dielectric constant due to the polarizability of the core electrons. In the above, a quadratic dispersion relationship is commonly used to extrapolate the optical dielectric function ε (ω, *q* = 0) onto the (ω, *q*)-plane:

4 $$\omega_{i}\;\left(q\right)=\omega_{i}\;\left(q=0\right)+\alpha q^{2}/2$$

where we chose α = 0, since the studied sample is an insulator, in accordance with the recommendation in ref 17.

To retrieve the optical constants, the DIIMFP (eq 1) normalized to unity area (nDIIMFP, red curve in Figure 3a) is fitted to the model dielectric function, eqs 3, by finding values for the oscillator parameters that minimize the chi-square deviation between the nDIIMFP and the experimental data. The resulting best fit is shown as the black curve in Figure 3a, and the corresponding values for the oscillator parameters are given in Table 2. The value for the static refractive index of *n*(0) = 1.54 is evaluated from these parameters.

**Table 2:** Parameters for the Extended Drude Model Dielectric Function for TinOAc; ε_b_ Was Taken to Be Unity

*A*_*i*_ (eV^2^)	Γ_*i*_ (eV)	ω_*i*_ (eV)
26.0	0.99	8.98
44.2	2.03	10.8
46.37	3.56	13.22
12.87	2.42	15.59
12.38	3.35	17.71
66.64	9.57	20.08
111.06	22.26	31.48
152.75	43.81	57.79

A test of the consistency of the extracted optical data is provided by checking the sum rules, in particular the *f*–sum rule, or Thomas-Reiche-Kuhn sum rule, which is given by^28^

5 f‐sum=12π2na∫0ωmaxω⁡Im[−1ε(ω,q=0)]dω

and must approach the average atomic number, *Z*_av_, for ω_max_ → ∞.

The Kramers–Kronig sum rule is given by

6 KK‐sum=2π∫0ωmax1ω⁡Im[−1ε(ω,q=0)]dω+1n(ω=0)2

and should approach unity for ω_max_ → ∞. Evaluation of the sum rules for the optical constants given in Table 2 yields *f*-sum = 5.89 and KK-sum = 0.98, in satisfactory agreement with the expected values *Z*_av_ = 5.88 and KK-sum = 1.00.

In Figure 4a, the photon absorption coefficient (i.e., the reciprocal absorption length) α = 2π*E*ε_2_/*hc*,^32^ derived from eqs 3, is compared with literature data in the XUV-EUV range.^14,22,29,30^ These data were all obtained from photoabsorption measurements and display reasonable mutual agreement. Note that the present data were obtained on a sample with well-defined composition and structure, whereas the literature data were obtained on a commercial material with undisclosed chemical structure.^30,31^ While the energy loss measurements are known to give accurate results in the single scattering range of energy losses (below ∼30 eV) deconvolution of the two REELS for larger energies introduces numerical uncertainty, and the present data are therefore less reliable in the EUV range. Nonetheless, the electron energy loss-derived data agree reasonably with the photoabsorption data for energies above ∼30 eV. The latter, on the other hand, are experimentally very challenging for smaller energies due to the large dynamic range of the absorptive part of the dielectric function, ε_2_, in this energy range. In view of these considerations, the agreement between the electron energy loss and photoabsorption data is satisfactory.

**Figure 4:**
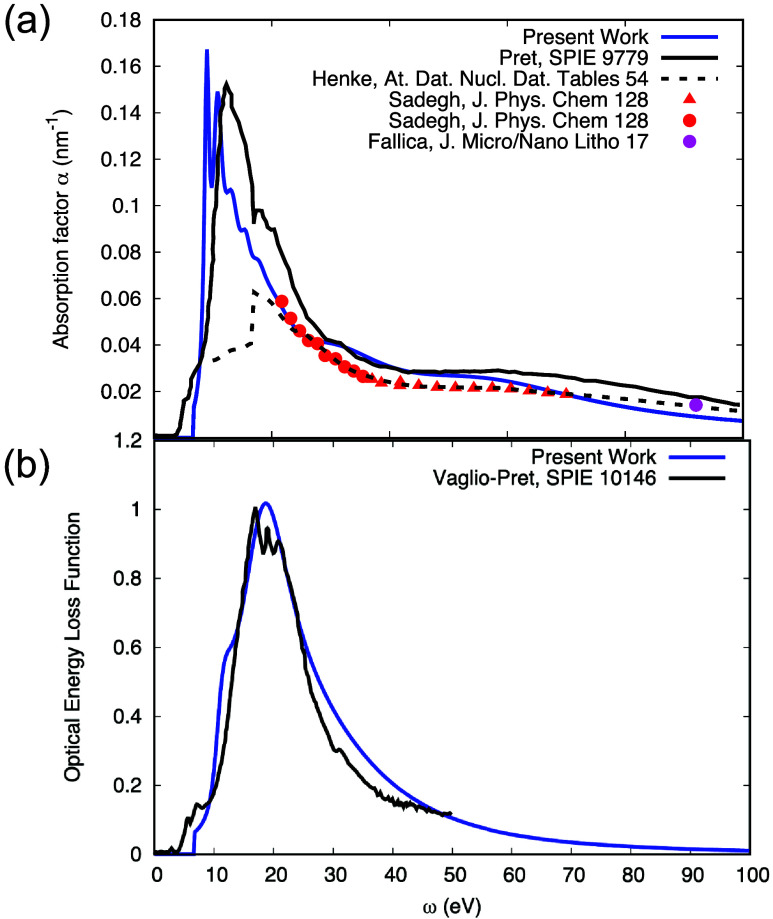
(a) Reciprocal absorption length α = 2π*E*ε_2_/*hc* obtained in the present work (blue), compared with data found in the literature.^14,22,29,30^ (b) Optical energy loss function, Im­{−1/ε­(ω, *q* = 0)}; present work (blue, dashed); data of Vaglio-Pret et al.^31^ (black).

Optical constants can be used to quantify not only photon absorption or photon beam attenuation but also the distance over which electron beams are attenuated, described by the electron IMFP. The IMFP is obtained from the energy loss function Im­{−1/ε­(ω, *q* = 0)} (ELF) by integration of eqs 1 over the allowed range of energy losses. The energy loss function derived here is compared with the data of Vaglio-Pret et al.^31^ in Figure 4b. A reasonable agreement is again observed, with the exception of the onset of energy losses (i.e., the HOMO–LUMO-distance or energy gap, *E*_g_), which is at a lower energy of about 4.9 eV in the literature data,^12^ while the onset of our ELF is at *E*_g_ = 6.6 eV (see below).

Figure 5 compares the characteristic length over which electron beams are attenuated, the IMFP, derived by numerical integration of eqs 1 over all allowed energy losses (dash-dotted black curve), with the predictive JTP formula by Jablonski, Tanuma, and Powell^33^ (red solid curve) for energies up to 5 keV. The JTP-values depend rather sensitively on the used value of the band gap. The dotted red curve is the JTP result assuming a band gap of 4.0 eV. For energies above 100 eV, we find good agreement between the JTP result and the present data, while the use of *E*_g_ = 4.0 eV in the JTP formula leads to slightly larger deviations.

**Figure 5:**
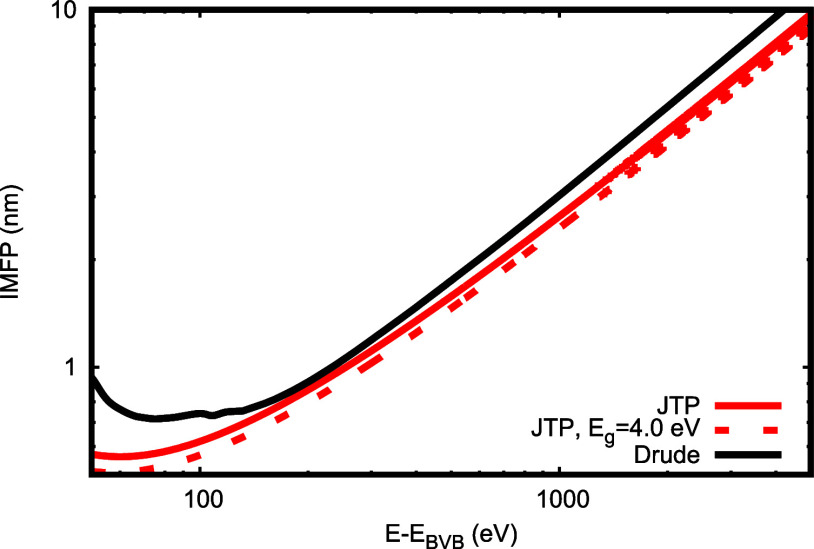
Red solid curve: Electron inelastic mean free path according to the predictive JTP formula (using E*g* = 6.6 eV);^33^ red dashed curve: JTP formula with *E*_g_ = 4 eV; IMFP calculated using direct integration of the DIIMFP over the energy loss (black dash-dotted curve).

The band gap and electron affinity can be determined from the onset of energy losses and coincidences, respectively. In the present work, the onset of bulk energy losses (in DIIMFP) is taken to be equal to the energy gap *E*_g_. Due to the presence of surface excitations, the onset of bulk losses in the DIIMFP is generally different from the onset of energy losses in a REELS, as is clearly seen in Figure 2a,b, where the blue filled curves representing surface excitations exhibit an earlier onset than the filled green curve, representing the bulk DIIMFP. This has been observed before,^16^ generally leading to bandgap values larger than those encountered in the literature. The red curve in Figure 3a represents the retrieved nDIIMFP, with an onset of *E*_g_ = 6.6 ± 0.5 eV.

Figure 3b shows the double differential secondary electron–electron energy loss coincidence spectrum (SE2ELCS) on a false color scale. The red parabola represents the bottom of the conduction band, *E*_2_ = Δ*E* – *E*_g_, the green parabola is described by *E*_2_ = Δ*E* – *E*_g_ – χ, i.e., the top of the valence band, the yellow parabola marks the bottom of the valence band *E*_BVB_. The intensity between Δ*E* = 10–25 eV corresponds to the region of the plasmon loss (cf. Figure 3a) with a maximum at about 20 eV. This feature in the coincidence spectrum is interpreted as excitation of a plasmon by an energy loss suffered by the primary electron; the plasmon subsequently decays, transferring its energy to a solid-state electron, which is then liberated and can escape as a secondary electron, provided it can overcome the surface barrier. Analysis of the coincidence data also yields information about the energy dissipation of slow electrons near solid surfaces,^21^ which will be presented elsewhere.

The onset of coincidences along the energy loss scale Δ*E*_coi_^min^, i.e., the smallest energy loss for which a secondary electron—created by indirectly absorbing the energy loss of the primary—can escape from the surface, is assumed to be equal to the energetic distance between the vacuum level and the valence band maximum (HOMO), Δ*E*_coi_^min^ = *E*_vac_ – *E*_VBM_. The electron affinity χ then follows as χ = Δ*E*_coi_^min^ – *E*_g_. Summing the coincidences over *E*_2_ yields the green curve in panel (a), from which the onset of coincidences is read off as χ + *E*_g_ = 9.9 ± 0.5 eV. This results in a value of χ = 3.3 ± 0.8 eV for the electron affinity. The width of the valence band is estimated on the basis of the coincidence spectrum as *E*_v_ = 8.5 eV, comparing reasonably with the estimate *E*_v_ = *a*_0_^2^ e^2^ (2π*ϱ*_v_)^2/3^/2 = 9.9 eV^34^ and findings by Zhang et al.^12^ The latter data, in combination with the band gap according to Vaglio-Pret et al.^31^ yields a result for the electron affinity of χ = 2.5 eV. Haitjema et al.^35^ determined the electron affinity in the gas phase (8.8 eV), while in the solid, it was found to be 3.5 ± 1 eV. The latter values both agree with our result within the experimental error.

## Conclusions

Analysis of electron energy loss spectra of a Tin-Oxo cage sample was performed yielding optical constants in the VIS-XUV range, and band parameters were determined by analysis of spectroscopy with single electrons and time-correlated electron pairs. The optical data are presented as parameters of an extended Drude-type model for the dielectric function. Physical quantities derived from them, such as the photon absorption factor, the energy loss function, and the IMFP, compare reasonably with literature data. A significant difference is found in the value of the energy gap, which is likely caused by differences in available states for optical and electronic transitions. The plasmon peak at 20 eV leads to an average of about three steps of energy loss of a primary photoelectron emitted from the valence band of the tin-oxo cage excited at an EUV energy of 92 eV. The total number of electron/hole pairs per photon is about 4 (three secondaries created in plasmon decay and one photoelectron). Concerning the discrepancy in the value of the electron affinity with earlier measurements, the difference between optical and electronic measurements of the band parameters will be further investigated in the future.
